# BMC Dermatology reviewer acknowledgement 2014

**DOI:** 10.1186/s12895-015-0020-3

**Published:** 2015-02-27

**Authors:** Hayley Henderson

**Affiliations:** BioMed Central, Floor 6, 236 Gray’s Inn Road, London, WC1X 8HB UK

## Abstract

The editors of BMC Dermatology would like to thank all our reviewers who have contributed to the journal in Volume 14 (2014).

**Ilknur Altunay**

Turkey

**Isabel Andia**

Spain

**Zoe Apalla**

Greece

**Alejandro Arenas-Pinto**

UK

**Giuseppe Argenziano**

Italy

**Wioletta Baranska-Rybak**

Poland

**Ioannis Bassukas**

Greece

**Robert Bransfield**

USA

**Robert Brodell**

USA

**Alex Burdorf**

Netherlands

**Ian Burgess**

UK

**Marco Castori**

Italy

**Hector Chinoy**

UK

**Elliot Coups**

USA

**Marian Currie**

Australia

**Soheil Dadras**

USA

**Robert Dellavalle**

USA

**Hoelzle Erhard**

Germany

**Stefano Fedele**

UK

**Steven Feldman**

USA

**Noriki Fujimoto**

Japan

**Georgios Gaitanis**

Greece

**Rebat Halder**

USA

**Pilar Iranzo**

Spain

**Takamichi Ito**

Japan

**Munenari Itoh**

Japan

**Feroze Kaliyadan**

Saudi Arabia

**Grazyna Kaminska-Winciorek**

Poland

**Jennifer Koplin**

Australia

**Chembolli Lakshmi**

India

**Peter Lio**

USA

**Marie Loden**

Sweden

**Ingrid Lundberg**

Sweden

**Eve Maubec**

France

**David Nelson**

USA

**Emiko Noguchi**

Japan

**Rei Ogawa**

Japan

**Anthony Ormerod**

UK

**Tae Hwan Park**

Korea, South

**Stefano Piaserico**

Italy

**Andor Pivarcsi**

Sweden

**Gaelle Quereux**

France

**Kristian Reich**

Germany

**June Robinson**

USA

**Laura Sawyer**

UK

**Fabiana Schuelter-Trevisol**

Brazil

**Daniel Shin**

USA

**Gregory Sonnenberg**

USA

**H. Peter Soyer**

Australia

**Wim Stevens**

Belgium

**Makoto Sugaya**

Japan

**Michael Sweeney**

USA

**Brian Tait**

Australia

**Andrés Tirado-Sánchez**

Mexico

**Hywel Williams**

UK

**Miriam Wittmann**

UK

**Xianyong Yin**

China

